# Transcription Factor SlAREB1 Is Involved in the Antioxidant Regulation under Saline–Alkaline Stress in Tomato

**DOI:** 10.3390/antiox11091673

**Published:** 2022-08-27

**Authors:** Zijian Xu, Fan Wang, Yongbo Ma, Haoran Dang, Xiaohui Hu

**Affiliations:** 1 College of Horticulture, Northwest AF University, Xianyang 712100, China; 2 Key Laboratory of Protected Horticultural Engineering in Northwest, Ministry of Agriculture, Xianyang 712100, China; 3 Shaanxi Protected Agriculture Research Centre, Xianyang 712100, China

**Keywords:** *Solanum lycopersicum*, transcription factor SlAREB, saline–alkaline stress, antioxidant enzymes

## Abstract

Basic leucine zipper (bZIP) transcription factors of the ABA-responsive element binding factor/ABA-responsive element binding proteins (ABF/AREB) subfamily have been implicated in abscisic acid (ABA) and abiotic stress responses in plants. However, the specific function of ABF/AREB transcription factors under saline–alkaline stress is unclear. Here, we identified four ABF/AREB transcription factors in tomato and found that SlAREB1 strongly responded to both ABA and saline–alkaline stress. To further explore the function of SlAREB1 under saline–alkaline stress, SlAREB1-overexpressing lines were constructed. Compared with wild-type plants, SlAREB1-overexpressing transgenic tomato plants showed reduced malondialdehyde content, increased the relative water content, and alleviated the degradation of chlorophyll under saline–alkaline stress. Importantly, SlAREB1 directly physically interacted with SlMn-SOD, which improved the activity of antioxidant enzymes and increased the scavenging of excess reactive oxygen species. Overall, the overexpression of SlAREB1 increased the antioxidant capacity of the transgenic tomato under saline–alkaline stress.

## 1. Introduction

Soil salinization has been increasing in recent years as a result of unsustainable farming practices and excessive fertilizer use, and such changes have severely limited the development of the vegetable industry. By the end of 2021, more than 8.7% of the world’s total land area (approximately 833 million hectares) was affected by soil salinization [1]. The long-term salinization of soil causes saline–alkaline stress, which inhibits plant growth and development [2]. Compared with neutral salt stress or alkaline stress alone, the combination of saline and alkaline stress tends to be more harmful to plants [3]. In addition to having the same osmotic stress, ion toxicity and oxidative stress as neutral salt stress, saline–alkaline stress also causes the harm of high pH stress [4,5]. Currently, the mechanisms of salt tolerance in plants under neutral salt stress have been extensively explored, whereas those of other salt types have not been thoroughly investigated [6,7].

During the growing phase, plants are exposed to a variety of adverse environmental factors and thus have developed resistance to certain stressors to survive. Plant hormones can regulate the development of resistance [8]. As one of the five major plant hormones, abscisic acid (ABA) plays a very important role in plant growth and stress responses [9]. Evidence has shown that the levels of endogenous ABA increase significantly in many plants under abiotic stresses, such as salinity, drought and low-temperature stress [10]. In tomato treated with NaCl, the content of endogenous ABA increases considerably [11]. To some extent, exogenous administration of ABA could mitigate the effects of salt stress on plants. By regulating the amounts of ions and organic solutes, exogenous ABA treatment at the right concentration could successfully promote the growth of wheat seedlings in salt and alkali stress conditions [12]. Our previous studies found that ABA was also involved in the response to saline–alkaline stress [13]. A pretreatment with ABA alleviated the degradation of chlorophyll in tomato seedlings under saline–alkaline stress, which further promoted the accumulation of proline and soluble sugars, reduced the content of reactive oxygen species (ROS), and improved the ability of antioxidant enzyme system. Furthermore, ABA-deficient mutants showed dramatically reduced tolerance to saline–alkaline stress [13].

Although the ABA signaling system has been shown to modulate plant adaptation to salt stress [14,15], the regulatory network of ABA signaling is extremely complex. In many studies, the interaction of protein phosphatase 2C (PP2C) and sucrose nonfermentation-related protein kinase 2 (SnRK2), two key regulators of the abscisic acid signaling pathway, is considered to be the most important regulatory mechanism [16,17,18]. SnRK2s are activated in the presence of ABA and then phosphorylate transcription factors (TFs) [19]. Subsequently, TFs that detect and bind to *cis* elements in the promoter regions upstream of their target genes directly activate ABA-responsive gene expression [20].

Among the transcription factors involved in the ABA signaling pathway, the AREB/ABFs are a major TFs class that encode basic-domain leucine zipper (bZIP) TFs, and they belong to a group-A subfamily that binds to ABA-responsive elements (ABREs, PyACGTGG/TC) in the promoter region of target genes [21,22,23]. Previous studies identified four AREB/ABF proteins (ABF1, AREB1/ABF2, ABF3, AREB2/ABF4) using the yeast one-hybrid screening method in *Arabidopsis* [24,25]. Among them, the *AREB1/ABF2*, *ABF3* and *AREB2/ABF4* genes are induced both by high salinity and drought, and they have been shown to be key regulators of ABA signaling in response to stress through genetic transformation analysis [26,27,28]. Although all three AREB/ABF TFs are induced by stress, only AREB1/ABF2 has been reported to be regulated by ABA-dependent phosphorylation [26,28]. In tomato, two AREB TFs (SlAREB1 and SlAREB2) were identified, and both were significantly induced by ABA treatment. However, it was found that SlAREB1 (but not SlAREB2) was mainly involved in the regulation of stress response-related gene expression [29,30]. Moreover, SlAREB1 has been reported to be a key factor in regulating ethylene biosynthesis by ABA during tomato fruit ripening [31]. Although AREB1 plays an important role in regulating the plant growth and development as well as the stress resistance, its regulatory mechanism under saline–alkaline stress needs to be further studied.

Tomato (*Solanum lycopersicum*) has emerged as a new model plant for biological studies because it represents one of the most widely cultivated vegetable crops in the world [32]. Here, we investigated the expression of the tomato *ABF/AREB* family under saline–alkaline stress, and we constructed *SlAREB1*-overexpression lines in the genetic background of wild-type in tomato cv. Ailsa Craig. The overexpression of *SlAREB1* in transgenic tomato plants significantly increases the tolerance to saline–alkaline stress and especially the antioxidant capacity. This research provides novel insights into exploring the antioxidant processes mediated by the ABA signaling pathway under saline–alkaline stress.

## 2. Materials and Methods

### 2.1. Plant Materials and Treatment

The CDS of *SlAREB1* gene (ID: Solyc04g078840) without terminator was cloned into the *pHellsgate8* vector. The wild-type (WT) tomato (*Solanum lycopersicum* L. cv. Ailsa Craig) was used as the genetic background. The tomato cotyledons were infected by the *Agrobacterium tumefaciens*-mediated transformation. The homozygous lines of *SlAREB1*-overexpression plants were obtained by continuous subculture, and the transgenic plants were detected by a PCR reaction (PrimeSTAR^®^ Max DNA Polymerase, Takara Bio, Shiga, Japan). Primers used in the study are presented in Table S3.

Seeds of WT and *SlAREB1*-overexpressing tomato were steeped in distilled water in a triangular flask and germinated in a shaker at 28 °C. The germinated seeds were planted in 50-well plates filled with organic stroma and cultured in an illumination incubator (RXZ-500D, Ningbo, China) under environmental conditions, which consisted of 25 °C/16 °C (day/night), 75–80% relative humidity, and a 12 h (photosynthetic photon flux density (PPFD), 350 μmol·m^−2^·s^−1^)/12 h (day/night) photoperiod. The seedlings showing fully developed their fourth true leaves were placed into seedling pots (one seedling per pot).

Each plant was irrigated with 100 mL of 300 mM saline–alkaline mixed solution (NaCl:Na_2_SO_4_:NaHCO_3_:Na_2_CO_3_ = 1:9:9:1, pH = 8.90) when the fifth true leaves were totally developed. Then, 100 mL distilled H_2_O was used to irrigate the control plants. For the ABA treatment, 100 μM ABA was evenly sprayed onto the leaves. The leaves from the second and third positions were gathered at 0, 1, 3, 6, 12, 24, and 48 h after the saline–alkaline treatment for gene expression analyses of the whole family of *SlABF/AREBs*, whereas the leaves used to determine other physiological indicators were collected after 7 days. All of the leaves were instantly frozen in liquid nitrogen and kept at –80 °C. To guarantee the reproducibility of the results, four independent experiments were carried out, each with three replicates.

### 2.2. Virus-Induced Gene Silencing (VIGS) of SlAREB1

The cDNA fragments of *SlAREB1* were amplified for gene silencing using primers listed in Table S3. Purified PCR products were ligated into the TRV2 vector. The resulting plasmids were transfected into the *Agrobacterium tumefaciens* strain carrying the helper vector TRV1; then, they were mixed and injected into WT tomato cotyledons to obtain the *SlAREB1*-genes silenced plants. Plants were maintained at 22 °C in an illumination incubator for three weeks before use. When the fifth true leaves of tomato seedling were completely expanded, qPCR was performed to ensure the silencing efficiency of approximately 30–40% of the TRV control plant transcript levels (Figure S4a).

### 2.3. Establishment of the Phylogenetic Tree and the Analysis of the Conserved Motif

The four *Arabidopsis* ABF/AREB protein sequences (ABF1, ABF2/AREB1, ABF3 and ABF4/AREB2) [33] were used to conduct a BLASTp query in the Phytozome database (http://www.phytozome.net/, accessed on 13 June 2022). We searched and identified the ABF/AREB protein sequences of eight plant species, including *Solanum lycopersicum*, *Solanum tuberosum*, *Cucumis sativus*, *Zea mays*, *Oryza sativa*, *Nicotiana tabacum*, *Vitis vinifera* and *Brassica rapa* (Table S1). A neighbor-joining Tree (N-J Tree) was established using MEGA7.0 software with full-length ABF/AREB protein sequences of nine plant species aligned by ClustalW [34]. Genetic distance was calculated by the Poisson calibration method and tested by 1000 bootstrap replications.

The conserved motifs of ABF/AREB proteins were analyzed using the Multiple Expectation Maximization for Motif Elicitation program (MEME version 5.4.1) (http://meme.nbcr.net/meme/cgi-bin/meme.cgi accessed on 25 June 2022) with the following parameters. The maximum number of motifs and the optimum motif width were set to 10 and 6–50 aa, respectively. To visualize the results, the final result was displayed using TBtools software (v1.098745, Guangzhou, China) [35].

### 2.4. Total RNA Extraction and RT–PCR Analysis

The Plant RNA extraction kit (OmegaBio-Tek, Doraville, GA, USA) was used to extract total RNA according to the manufacturer’s instructions. The PrimeScript^TM^ RT reagent Kit (Takara Bio, Shiga, Japan) was used to perform reverse transcription for single-stranded DNA synthesis according to the manufacturer’s instructions. Quantitative reverse transcription-polymerase chain reaction (qRT–PCR) was used to measure gene expression. To normalize gene expression, the *SlActin* gene (Gene ID: Solyc03g078400.3) was chosen as the internal control. qRT–PCR was performed using StepOne Software v2.3 (Applied Biosystems, Waltham, CA, USA) and ChamQ SYBR qPCR Master Mix (Vazyme, Nanjing, China). The 2^−ΔΔCt^ technique was used to perform the gene expression fold change analysis [36]. PCR primers prepared by Primer Premier 5 and mentioned in Table S2 were used to amplify the transcripts.

### 2.5. Analysis of the Plant Damage Physiological Parameters: Relative Water Content, Chlorophyll Fluorescence and Content

Relative water content (RWC) was measured according to the method described by Hu et al. [37]. Briefly, to determine RWC, five leaves were weighed to determine the fresh weight (FW). Then, the leaves were completely immersed in distilled water for 8 h and dried to measure the saturated fresh weight (SFW). Subsequently, the leaves were placed in a 65 °C oven for drying and weighted to determine the dry weight (DW). The RWC was evaluated as follows: $RWC=\frac{FW-DW}{SFW-DW}\times 100\%$.

Chlorophyll fluorescence was measured using the Open FluorCam FC 800-O system (PSI, Photon Systems Instruments, Brno, Czech Republic) according to the parameters of Xu et al. [13] and calculated using Fluorcam7 software (PSI, Brno, Czech Republic). The ratio of the variable fluorescence to maximum yield of fluorescence was used to express the maximal photochemical efficiency (*Fv/Fm*) of PSII.

The chlorophyll and carotenoids contents were extracted with 80% acetone for one night and measured at 470 nm, 645 nm and 663 nm. The chlorophyll and carotenoids contents were calculated as described by Xu et al. [13].

### 2.6. Analysis of the Plant Damage Oxidative Parameters: Lipid Peroxidation, H_2_O_2_ and O_2_^−^ Contents, Superoxide Dismutase (SOD) Activity and Total Antioxidant Capacity

Lipid peroxidation is attained by the measurement of malondialdehyde (MDA) content. MDA content was measured as previously described [38]. Leaf frozen samples (0.2 g) were powdered using a high-throughput tissue lyser (Scientz, Ningbo, China) in liquid nitrogen with 1.8 mL of 5% (*w*/*v*) trichloroacetic acid and then vortexed and centrifuged at 12,000× *g* for 20 min. Subsequently, 1.5 mL of supernatant was added to 1.5 mL of 0.6% thiobarbituric acid, which was boiled (100 °C) for 30 min and then reduced to room temperature to measure the absorbance at 450 nm, 532 nm and 600 nm. The MDA content was evaluated as follows: $MDA=6.453\times \left(A_{532}-A_{600}\right)-0.559\times A_{450}$.

The H_2_O_2_ and O_2_^−^ contents in the tomato leaves were measured spectrophotometrically based on the methods of Sui et al. [39] and Wang and Luo [40], respectively. The H_2_O_2_ level was determined at the absorbance at 415 nm according to the standard curve plotted with a known concentration, while the O_2_^−^ content was calculated per gram of fresh mass of leaves at 530 nm. The accumulation of H_2_O_2_ and O_2_^−^ was examined by histochemical staining with 3,3′-diaminobenzidine (DAB) and nitro blue tetrazolium (NBT) respectively [41]. Briefly, tomato leaves were placed in a solution of 1 mg mL^−1^ DAB (pH 3.8) for 8 h under light to analyze the H_2_O_2_ and in 0.5 mg mL^−1^ NBT for 8 h in the dark to analyze the O_2_^−^.

The SOD activity was detected by ultraviolet spectrophotometry in accordance with the manufacturer’s protocols through corresponding test kits (for plants) purchased from Solarbio^®^ Life Sciences (Beijing, China). The Mn-SOD activity was measured by a Multifunctional Enzyme Marker (Infinite M200 Pro, Tecan, Germany) according to the instructions of the Cu/Zn-SOD and Mn-SOD Assay Kit with WST-8 (Beyotime Biotechnology, Shanghai, China), and it was detected at a wavelength of 450 nm.

The total antioxidant activity of tomato leaves was determined by a ferric-reducing ability of plasma (FRAP) assay [42]. A high-throughput tissue lyser (Scientz, Ningbo, China) was used to powder 0.2 g of frozen tomato leaves, and the powder was then combined with 2.5 mL of methanol for 2 h and centrifuged at 10,000× *g* for 10 min at room temperature. The supernatant (0.1 mL) was added to 0.3 mL distilled water and 3.0 mL of tripyridine triazine (TPTZ) reagent, which was then incubated at 37 °C for 10 min. Then, the absorbance of each sample at 593 nm was measured [43]. The antioxidant capacity is expressed as mmol FeSO_4_ equivalents per gram of sample (FW).

### 2.7. Yeast Two-Hybrid (Y2H) Assay

The full-length CDS of the *SlMn-SOD* gene was amplified with tomato cDNA as the template and recombined into the pGADT7 vector to construct the prey vector. Fragments of *SlAREB1* with different lengths were intercepted and inserted into the pGBKT7 vector to construct the bait vectors. The target fragments were amplified by PCR primers that were designed by Primer Premier 5 and are listed in Table S3. The prey and bait vectors were co-transferred into the Y2H Gold yeast strain as described in the instructions (Clontech, Shanghai, China). Yeast strains carrying the indicated vectors were plated on SD (−T/−L) growth medium. The positive clones were diluted with 0.9% NaCl to an OD_600_ of 0.2, after which 10 μL of each suspension was dropped on SD (−T/−L), SD (−T/−L/−H/−A) and SD (−T/−L/−H/−A with X-α-Gal) medium.

### 2.8. Bimolecular Fluorescence Complementation (BiFC) Assays

The full-length CDSs of the *SlMn-SOD* and *SlAREB1* genes were amplified and inserted into the nYFP and cYFP plasmids, respectively. The resulting plasmids were cotransfected into *Agrobacterium tumefaciens* strain GV3101 as previously described [44]. The tobacco leaves were then examined for yellow fluorescent protein (YFP) expression using a Leica SP8 confocal laser scanning microscope after transformation for 36–48 h. The nuclei were stained with DAPI as a control.

### 2.9. Statistical Analysis

Data represent the average of three independent experiments and are shown as the means ± SDs. The statistical analysis was performed using GraphPad Prism 8.0 software (GraphPad Software, Waltham, CA, USA) and SPSS software (IBM SPSS statistics 22.0, New York, NY, USA). Statistically significant differences were determined by subjecting the data to one-way ANOVA with Tukey’s multiple range test (*p* < 0.05 or *p* < 0.01).

## 3. Results

### 3.1. Phylogenetic and Conserved Motif Analyses of SlABF/AREBs

**Figure 1:**
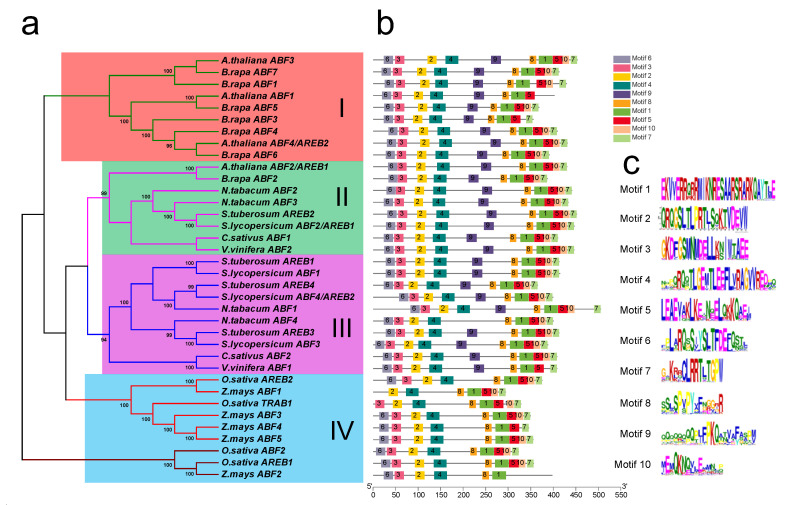
The phylogenetic tree and conserved motif analyses of SlABF/AREB proteins. (**a**) The phylogenetic tree of ABF/AREB proteins in 9 plants, including *Solanum lycopersicum*, *Solanum tuberosum*, *Cucumis sativus*, *Zea mays*, *Oryza sativa*, *Nicotiana tabacum*, *Vitis vinifera* and *Brassica rapa*. (**b**) Conserved domains of the ABF/AREBs were analyzed and shown by MEME. (**c**) Sequence logos of the conserved motifs displayed by WebLogo.

To investigate the evolutionary relationship of tomato ABF/AREB family proteins, a neighbor-joining (NJ) phylogenetic tree was constructed with ABF/AREB proteins from nine typical plants, including monocots and dicotyledons (Figure 1a). Based on the phylogenetic tree, the ABF/AREB proteins were grouped into four groups (I, II, III and IV). Four tomato ABF/AREB proteins were located in groups II and III, and the monocots were placed into group IV. Furthermore, the conserved motifs were evaluated using the MEME tool, which identified a total of 10 motifs in all the ABF/AREBs (Figure 1b,c). The basic leucine zipper domain of motif 1 was present in all the ABF/AREB proteins. Compared with the dicotyledons, the monocots commonly lacked motif 9. We further analyzed the protein length, molecular mass, and pI values of all ABF/AREB proteins (Table S1). According to our results, the length and molecular mass of ABF/AREBs ranged from 270 to 506 amino acid residues and 30.51 to 55.73 kD, respectively, with a mean of 393 amino acid residues and 42.68 kD. Tomato AREB1 belongs to group II, and it is the longest and largest ABF/AREB (447 amino acid residues and 47.98 kD) in tomato. The pI value of ABF/AREBs ranged from 5.44 to 9.92. ABF/AREBs from monocots displayed a tendency to maintain acidic pI values, while dicotyledons tend to show more alkaline values (Table S1).

### 3.2. Expression Analysis of SlABF/AREB Genes

**Figure 2:**
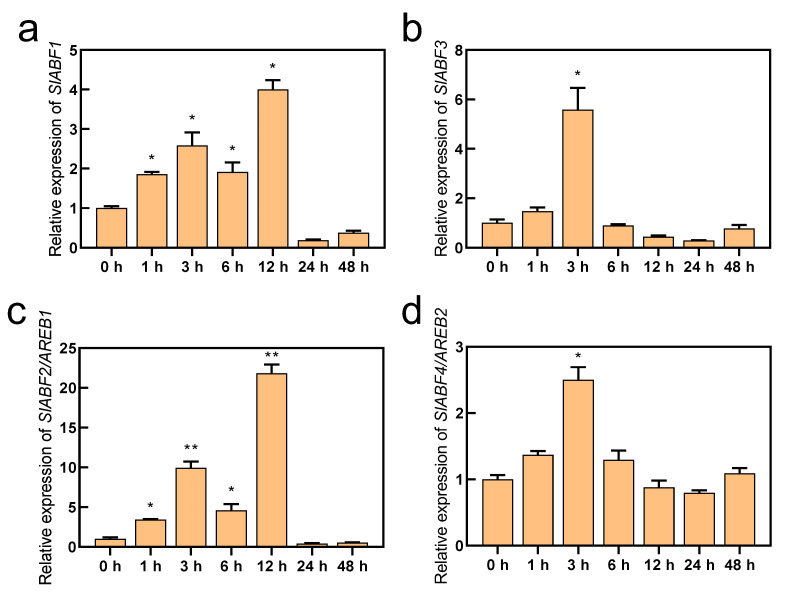
The expression of *SlABF/AREB* subfamily genes with ABA treatment. The wild-type tomato seedlings with fifth true leaves were treated with 100 μM ABA for 0, 1, 3, 6, 12, 24 and 48 h. The expression of (**a**) *SlABF1*, (**b**) *SlABF3*, (**c**) *SlABF2/AREB1* and (**d**) *SlABF4/AREB2* genes was measured. *SlACTIN* was used as an internal control. The relative expression of genes was based on ABA treatment for 0 h. Values represent averages of four independent measurements, and error bars represent standard errors. Asterisks denote a significant difference compared with the control (* *p* < 0.05, ** *p* < 0.01).

**Figure 3:**
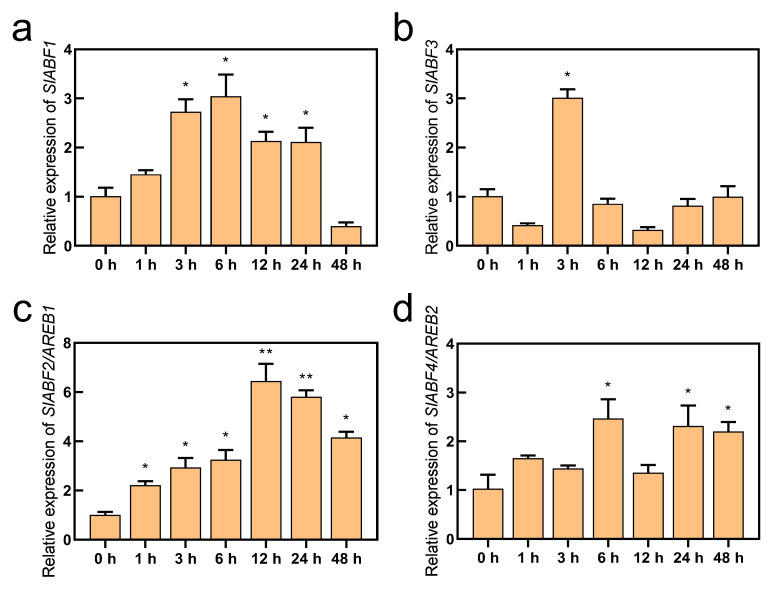
The expression of *SlABF/AREB* subfamily genes under saline-alkaline stress. The wild-type tomato seedlings with fifth true leaves were treated with 300 mM saline–alkaline mixed scheme for 0, 3, 6, 12, 24 and 48 h. The expression of (**a**) *SlABF1*, (**b**) *SlABF3*, (**c**) *SlABF2/AREB1* and (**d**) *SlABF4/AREB2* genes was measured. *SlACTIN* was used as an internal control. The relative expression of genes was based on saline–alkaline treatment for 0 h. Values represent averages of four independent measurements, and error bars represent standard errors. Asterisks denote a significant difference compared with the control (* *p* < 0.05, ** *p* < 0.01).

ABF/AREBs have been found to respond to a variety of abiotic stressors, including drought and salt stress, and they represent essential transcription factors in the ABA signaling pathway [28,45,46]. In this study, we obtained the RPKM values of *SlABF/AREBs* in the different tissues of tomato using the Tomato Functional Genomics Database (http://ted.bti.cornell.edu/ accessed on 10 July 2022). Except for SlABF3, the findings demonstrated that the expression of the *SlABF/AREB* genes varied noticeably among tomato tissues. Although the fruits exhibited the highest levels of *SlABF1*, *SlAREB1*, and *SlAREB2* expression, these genes were also expressed in the leaves and roots (Figure S1). Furthermore, all four identified *SlABF/AREB* genes were induced by ABA (Figure 2). However, their expression levels were greatly different. Although *SlABF1*, *SlABF3* and *SlAREB2* were upregulated by the ABA treatment, their expression intensities were markedly lower than that of *SlAREB1*. Similarly, four *SlABF/AREB* genes were also induced by saline–alkaline stress (Figure 3). The expression levels of *SlAREB1* in the leaves were rapidly induced by saline–alkaline treatment after 1 h with a maximum 6-fold increases observed at 12 h, whereas the expression of *SlABF1*, *SlABF3* and *SlAREB2* increased only approximately 3-fold over the whole time period. These results suggest that SlAREB1 has a predominant role in regulating ABA-mediated saline–alkaline stress responses in tomato.

### 3.3. Overexpression of SlAREB1 Results in Saline–Alkaline Tolerance in Tomato Seedlings

**Figure 4:**
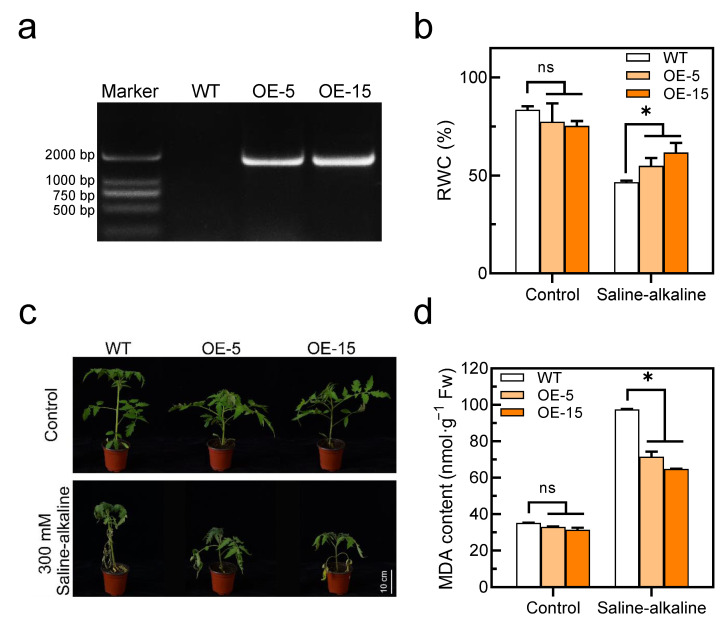
Overexpression of SlAREB1 confers enhanced saline–alkaline tolerance in transgenic tomatoes. (**a**) Confirmation of the presence of the transgene in SlAREB1-overexpression lines by PCR analysis. (**b**) The relative water content of wild-type (WT) and transgenic (OE-5 and OE-15) tomato leaves with 300 mM saline–alkaline mixed solution for 7 d. (**c**) Phenotype of tomato seedlings under 300 mM saline–alkaline stress in WT and transgenic tomatoes. (**d**) The MDA content of WT and transgenic tomatoes with 300 mM saline–alkaline mixed solution for 7 d. Distilled water was used as control. Values represent averages of four independent measurements, and error bars represent standard errors. Asterisks denote a significant difference compared with the control (* *p* < 0.05).

**Figure 5:**
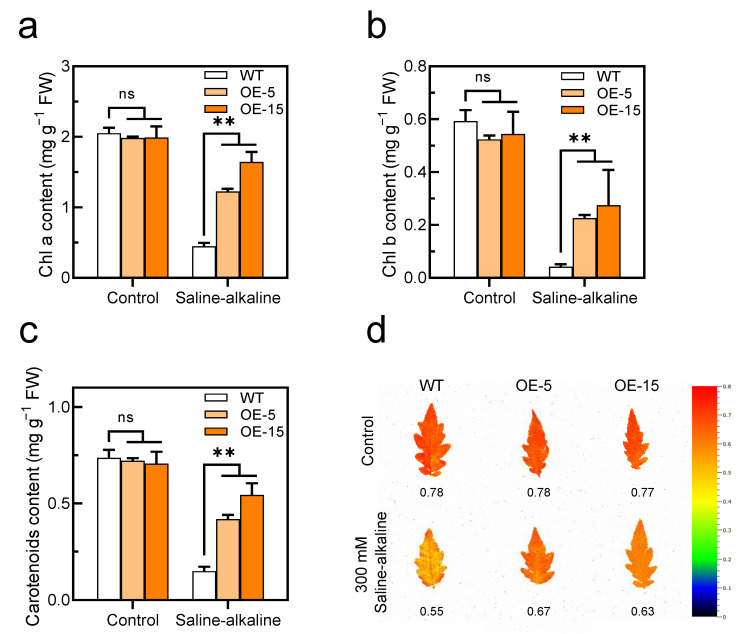
Overexpression of SlAREB1 confers enhanced photosynthetic capacity in transgenic tomatoes under saline–alkaline stress. (**a**–**c**) Chlorophyll a, chlorophyll b and carotenoids contents and (**d**) chlorophyll fluorescence (*Fv/Fm*) were measured in wild-type (WT) and transgenic (OE-5 and OE-15) tomato seedlings after 7 d of treatment with 300 mM saline–alkaline mixed solution. Distilled water was used as control. Values represent averages of four independent measurements, and error bars represent standard errors. Asterisks denote a significant difference compared with the control ( ** *p* < 0.01).

Since *SlAREB1* was strongly upregulated by saline–alkaline stress, *SlAREB1*-overexpression lines were used to investigate whether this transcription factor is involved in saline–alkaline stress. We constructed two SlAREB1-overexpression lines and confirmed the presence of the transgene in SlAREB1-OE tomato seedlings by PCR (Figure 4a). Under normal growth conditions, morphological differences could not be discerned between the tomato transgenic lines (OE-5 and OE-15) and wild-type (WT). Transgenic lines were impacted less severely than the WT after a 7-day treatment with 300 mM saline–alkaline, which caused obvious leaf damage (Figure 4c). Without the saline–alkaline stress, the RWC and MDA content were similar in the WT and transgenic lines, whereas after the saline-alkaline treatment, the RWC in the WT was significantly lower than that in the transgenic lines, and the MDA content was remarkably higher in the WT than in the transgenic lines (Figure 4b,d). In contrast, the MDA content in the *SlAREB1*-silenced tomato lines was significantly higher than in the control (TRV2-empty) under saline-alkaline stress (Figure S4b). Moreover, the Chl content and Chl fluorescence (*Fv/Fm*) were indistinguishable between the WT and transgenic lines before the saline–alkaline treatment. Saline–alkaline stress decreased the Chl content and *Fv/Fm* ratios in all genotypes, although that of the WT was significantly lower than that of the transgenic lines (Figure 5). These results indicate that the overexpression of *SlAREB1* enhances saline–alkaline tolerance in transgenic tomato.

### 3.4. Overexpression of SlAREB1 Enhances the ROS Scavenging Capacity

**Figure 6:**
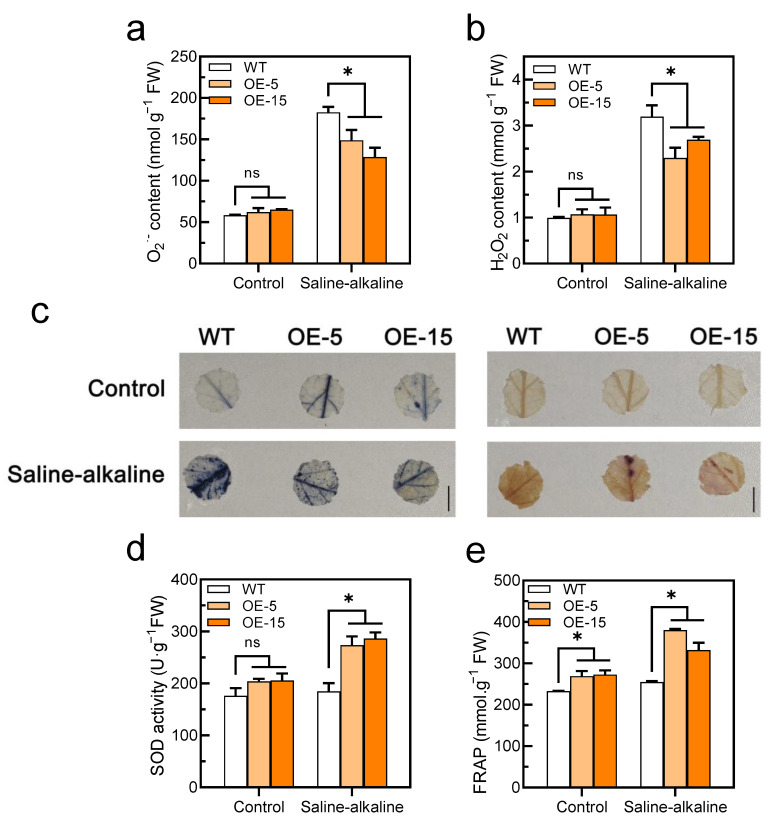
Overexpression of SlAREB1 confers enhanced the antioxidant capacity in transgenic tomatoes under saline–alkaline stress. (**a**) O_2_^−^ and (**b**) H_2_O_2_ contents after 7 d of treatment with 300 mM saline–alkaline mixed solution in wild-type (WT) and transgenic (OE-5 and OE-15) tomato seedlings. (**c**) In situ accumulation of O_2_^−^ (blue) and H_2_O_2_ (brown) of WT and transgenic tomatoes, as revealed by histochemical staining with nitro blue tetrazolium (NBT) and 3,3′-diaminobenzidine (DAB), respectively, under saline–alkaline treatment. (**d**) SOD activity and (**e**) total antioxidant capacity (FRAP) in WT and transgenic tomatoes under saline–alkaline stress for 7 d. Distilled water was used as control. Values represent averages of four independent measurements, and error bars represent standard errors. Asterisks denote a significant difference compared with the control (* *p* < 0.05).

Significant differences in ROS (O_2_^−^ and H_2_O_2_) contents were not observed in the tomato leaves between the WT and transgenic lines without the saline–alkaline treatment. However, the ROS contents were increased in both genotypes under saline–alkaline stress, although the accumulation in the transgenic lines was remarkably lower than that in the WT (Figure 6a,b). Similarly, the accumulation of O_2_^−^ and H_2_O_2_ in the two genotypes was examined in situ by histochemical staining with NBT and DAB, respectively. The results showed that the WT had more ROS than the transgenic lines under saline–alkaline treatment (Figure 6c). However, there was no significant difference in O_2_^−^ and H_2_O_2_ content between WT and *SlAREB1*-silenced tomato lines under saline–alkaline stress (Figure S4d,e). Without the saline–alkaline treatment, although the SOD activity was a slightly higher than that of the WT in the transgenic lines, discernible differences were not observed between the two genotypes. However, the SOD activity was noticeably higher in the transgenic lines than the WT under saline–alkaline stress (Figure 6d), but the *SlAREB1*-silenced lines showed no different than the WT (Figure S4c). Although the SOD activity of the WT did not significantly increase under saline–alkaline stress, the expression of the *SlSOD* gene was remarkably induced (Figure S2a). Similarly, the total antioxidant capacity (FRAP) of the transgenic lines was significantly higher than that of the WT after the saline–alkaline treatment (Figure 6e). These results confirmed that the overexpression of *SlAREB1* improved the antioxidant capacity and enhanced the ROS scavenging in tomato seedlings.

### 3.5. SlAREB1 Directly Interacts with SlMn-SOD

**Figure 7:**
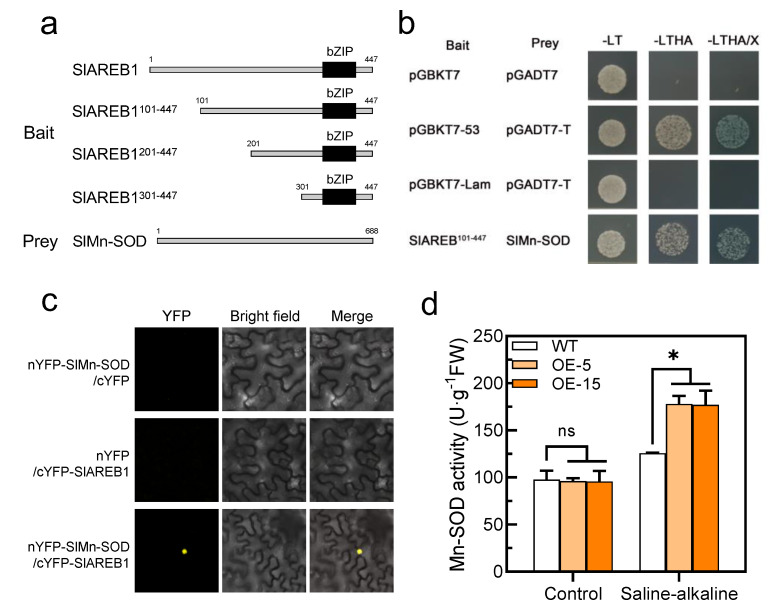
SlAREB1 directly interacts with SlMn-SOD. (**a**) Schematic diagram of the SlAREB1-truncated fragments used to construct the bait vectors. Amino acid positions of fragments were numbered. (**b**) SlAREB1 interaction with SlMn-SOD as determined in yeast two-hybrid assays. (**c**) SlAREB1 interaction with SlMn-SOD in vivo as determined by bimolecular fluorescence complementation (BiFC) assay. Yellow mark indicates fluorescence signals. (**d**) Mn-SOD activity in WT and transgenic tomatoes under saline–alkaline stress for 7 d. Distilled water was used as control. Values represent averages of four independent measurements, and error bars represent standard errors. Asterisks denote a significant difference compared with the control (* *p* < 0.05).

Since the SOD activity was significantly increased in the transgenic lines under saline–alkaline stress, we speculated that the transcription factor SlAREB1 may regulate the transcriptional level of the *SlSOD* gene. However, we found no differences in the expression of the *SlSOD* genes between the WT and transgenic lines (Figure S2b). Furthermore, the SlAREB1-truncated fragments were used to construct the bait vectors (Figure 7a). The longest fragment without self-activation was used to screen the cDNA library on the screening medium (Figure S3). We identified SlMn-SOD as an SlAREB1-interacting protein via Y2H analysis. As shown in Figure 7b, the yeast cells expressing both BD-SlAREB1^101–447^ and AD-SlMn-SOD grew on the selection medium. Using a bimolecular fluorescence complementarity (BiFC) assay, we further confirmed the interaction of SlAREB1 and SlMn-SOD in vivo (Figure 7c). Moreover, the additional experiment indicated that the overexpression of SlAREB1 positively regulated the activity of Mn-SOD under saline–alkaline stress in tomato seedlings (Figure 7d). These results demonstrated that SlAREB1 could directly interact with SlMn-SOD and enhance its activity.

## 4. Discussion

The transcription factors ABF/AREBs, which are important in the ABA signaling pathway, have recently received much attention [20]. These transcription factors have been identified and studied in *Arabidopsis* [25], tobacco [47], soybean [48] and wheat [3]. In tomato, we identified four ABF/AREB transcription factors and analyzed the conserved motifs (Figure 1). The most conserved Motif 1 was the bZIP domain which was found in all nine species. Moreover, we found that Motif 9 was present only in dicotyledons, which may be a specific motif associated with dicotyledon evolution [12]. Two previously identified SlAREB transcription factors in tomato were induced by ABA, although only SlAREB1 was strongly stimulated in response to salt and drought stress [29]. However, in this study, four *SlABF/AREB* genes were induced by ABA and saline–alkaline conditions (Figure 2 and Figure 3). Among them, the expression level of *SlAREB1* was most strongly induced. Therefore, the *SlAREB1* gene was selected to construct transgenic lines, and its gene function was studied under saline–alkaline stress.

A large number of studies have shown that ABF/AREB transcription factors play an important role in a variety of stress adaptations and resistance. ABA-dependent modification is necessary for the full activation of all ABF/AREB transcription factors in rice and *Arabidopsis* [28]. Compared to WT plants, transgenic *Arabidopsis* plants overexpressing AREB1 have higher ABA sensitivity and drought tolerance, and the overexpression of AREB1 enhances the expression of ABA regulatory genes [26]. In tomato, SlAREB1 is expressed mainly in the leaves, roots and flowers, whereas the transcript levels are barely detected in the mature seeds and red fruits [49]. However, our results show that the tomato ABF/AREB-type transcription factors (except for SlABF3) are expressed not only in the leaves, roots and flowers but also in the fruits (Figure S1). The overexpression of *SlAREB1* increased the resistance of transgenic tomatoes to salt and drought stress and decreased chlorophyll fluorescence in drought and lipid peroxidation damage in salt stress [29]. Here, the overexpression of *SlAREB1* similarly increased the saline–alkaline tolerance in tomato seedlings (Figure 3c).

In our previous research, saline–alkaline stress caused cell membrane lipid peroxidation in tomato seedlings. After the cell membrane was disrupted, the non-selective permeability of the plasma membrane is lost; thus, a significant amount of electrolyte is leaked to the extracellular space, and therefore, the RWC of leaves decreased [13]. This study demonstrated once more that saline–alkaline stress increases the MDA content and decreases RWC in tomato leaves. However, the accumulation of MDA in the transgenic lines was less than that in the WT, while the RWC was higher (Figure 4b,d). Moreover, the chlorophyll content of tomato leaves decreased significantly under saline–alkaline stress. The degradation of chlorophyll reduces photosynthetic capacity, which has a negative impact on plant growth and development [50]. Here, our study found that the overexpression of *SlAREB1* effectively reduced the degradation of chlorophyll and increased the ratio of chlorophyll fluorescence in transgenic tomatoes under saline–alkaline stress (Figure 5). Overall, SlAREB1 has a positive effect on improving the cell membrane integrity and photosynthesis of tomato seedlings under saline–alkaline stress.

Plants with aerobic metabolism must produce ROS, which are key signaling molecules that regulate abiotic stress responses and physiological responses in plants. However, excessive concentrations of ROS are able to stunt plant development or even cause plant death [51,52]. Under saline–alkaline stress, ROS (O_2_^−^ and H_2_O_2_) accumulate excessively in tomato leaves, resulting in oxidative stress [13]. Nevertheless, the accumulation of ROS in *SlAREB1*-overexpressing lines was obviously reduced compared with that in the WT under stress (Figure 6a–d). Plants maintain normal physiological activities in the presence of excessive ROS through the scavenging effect of antioxidant defense systems [53]. The antioxidant enzyme system is considered to be the most important defense system of plants under oxidative stress. The antioxidant enzyme system in plants mainly includes superoxide dismutase (SOD), catalase (CAT), peroxidase (POD) and ascorbate peroxidase (APX) [54,55,56]. SOD proteins, as an antioxidant enzyme, play critical roles in plant responses to various environmental stresses [54]. Short-term saline–alkaline stress induced the expression of the *SlSOD* gene (Figure S2a), although the activity of SOD was not different in the WT under long-term saline–alkaline stress. In contrast, the SOD activity was significantly increased in the transgenic lines (Figure 6d). Thus, the transgenic lines presented a stronger antioxidant capacity than the WT tomato seedlings (Figure 6e). These results further indicate that *SlAREB1* overexpression led to stronger tolerance under saline–alkaline stress in tomato seedlings.

Although ABA has been observed to enhance SOD activity [57,58], its potential regulatory mechanism is still unknown. Here, it was discovered that SlAREB1, a crucial transcription factor in the ABA signaling pathway, increased SOD activity when exposed to saline–alkaline stress (Figure 6d). Therefore, we hypothesize that SlAREB1 may be involved in regulating the transcription of *SlSOD* genes. However, there was no significant difference in the expression level of *SlSOD* between the two genotypes (Figure S2b). Moreover, we used SlAREB1 as bait to screen the yeast cDNA library, and we identified SlMn-SOD. Mn-SOD is a crucial factor that eliminates ROS in plant cells under environmental stress, and transgenic *Arabidopsis* overexpressing Mn-SOD showed improve salt tolerance [59]. Next, we further confirmed that SlAREB1 physically interacts with SlMn-SOD in vitro and in vivo and that it positively regulates the Mn-SOD activity in response to saline–alkaline stress (Figure 7). In conclusion, our results expand the novel regulatory network for SlAREB1 in saline–alkaline tolerance and enrich data on the ABA signaling pathway under saline–alkaline stress.

## Data Availability

Data are contained within the article and supplementary material.

## Supplementary Materials

The following supporting information can be downloaded at: https://www.mdpi.com/article/10.3390/antiox11091673/s1, Table S1: ABF protein properties and their clade-distributions in land plants; Table S2: Primers used for quantitative real-time PCR; Table S3: List of primers used in this study; Figure S1: The RPKM values of SlABF/AREBs in the different tissues of tomato; Figure S2: The expression level of SlSOD gene; Figure S3: Self-activation validation of the SlAREB1-truncated fragments used for yeast cDNA library screening; Figure S4: Analysis of the plant damage oxidative parameters in SlAREB1 gene silenced lines with saline–alkaline stress.
