# Supplementary material for: Transcription Factor SlAREB1 Is Involved in the Antioxidant Regulation under Saline–Alkaline Stress in Tomato
Source: Antioxidants (Basel). 2022 Aug 27;11(9):1673. doi: 10.3390/antiox11091673 (PMC9495317; doi:10.3390/antiox11091673)
Supplement: Supplementary file 1 [file antioxidants-11-01673-s001.zip › Supplementary figure.pdf]

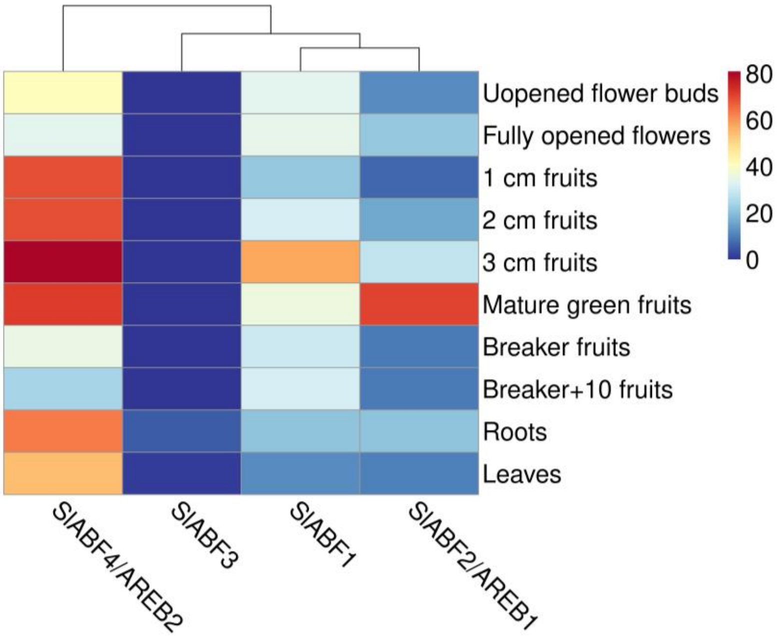

Figure S1 The RPKM values of *SlABF/AREBs* in the different tissues of tomato. The RPKM values were obtained from Tomato Functional Genomics Database (http://ted.bti.cornell.edu/).

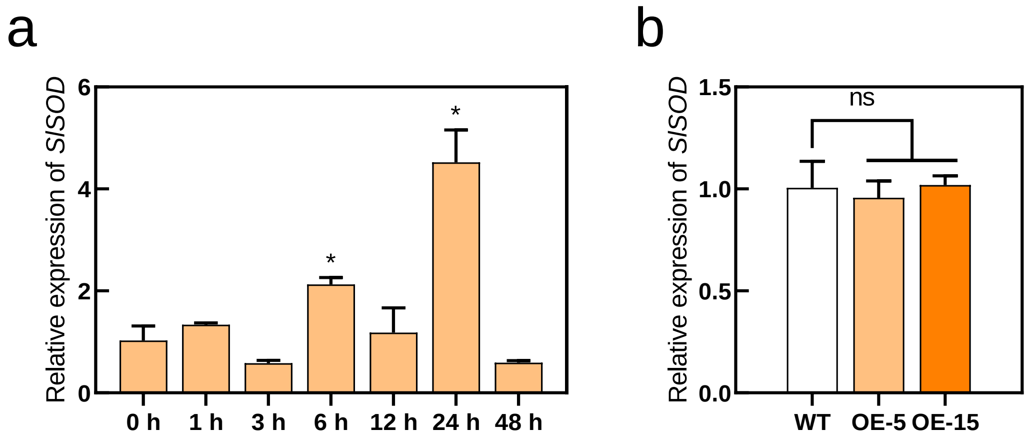

Figure S2 The expression level of *SlSOD* gene. (a) Expression of *SlSOD* in wild-type tomato seedlings with 300 mM saline-alkaline stress for 0, 1, 3, 6, 12, 24 and 48 h. The relative expression of genes was based on saline-alkaline treatment for 0 h. (b) Expression of *SlSOD* in wild-type (WT) and transgenic (OE-5 and OE-15) tomatoes. The relative expression of genes was based on WT. Values represent averages of four independent measurements, and error bars represent standard errors. Asterisks denote a significant difference compared with the control (*$P < 0.05$).

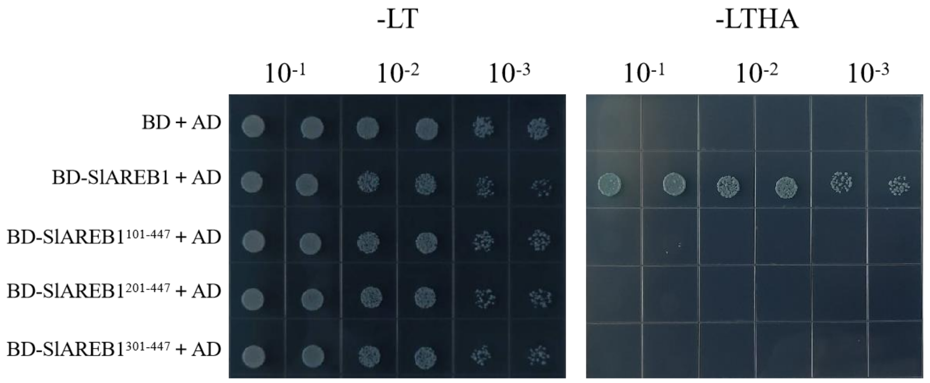

Figure S3 Self-activation validation of the SlAREB1-truncated fragments used for yeast cDNA library screening. The SlAREB1-truncated fragments were used to construct the bait vectors and co-transferred into Y2H Gold yeast strain with pGADT7-empty. The validation was performed on SD (-T/-L) and SD (−T/−L/−H/−A) medium.

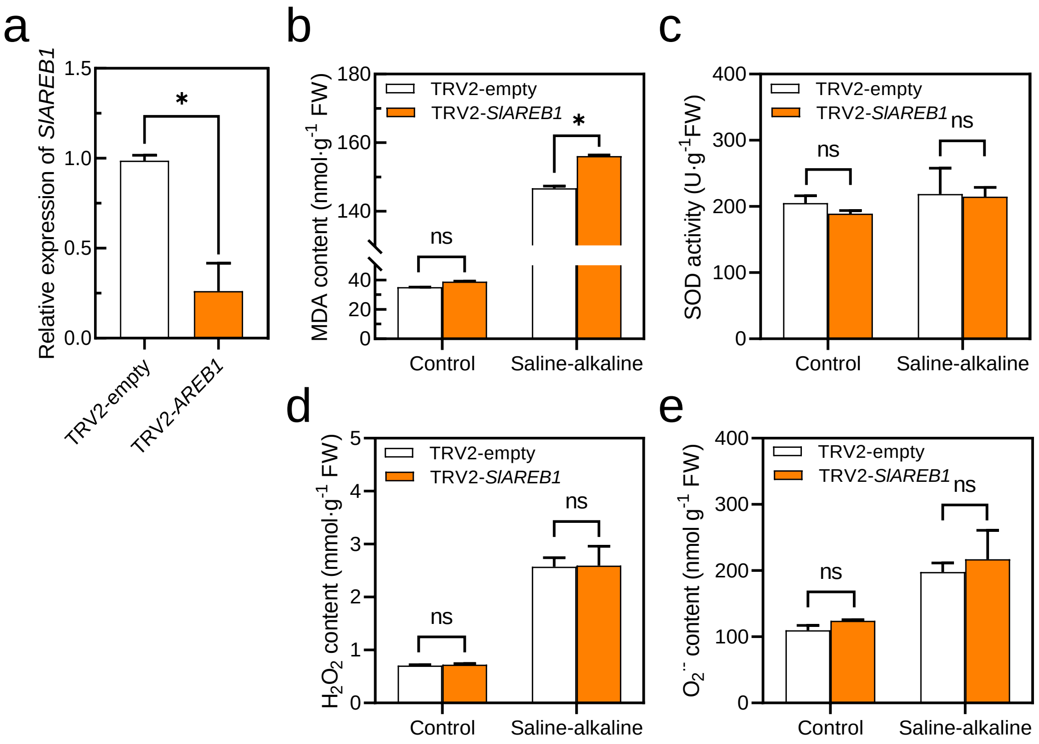

Figure S4 Analysis of the plant damage oxidative parameters in *SlAREB1* gene silenced lines with saline-alkaline stress. (a) The *SlAREB1* gene was silenced in 'Ailsa Craig' background tomato, and *SlAREB1* gene expression was analyzed at 30 days after *Agrobacterium*-infection VIGS plants. The expression of genes was based on TRV2-empty tomato. (b) MDA content, (c) SOD activity, (d) H_2O_2 and (e) $O_2^{\cdot -}$ content were measured in silenced plants after 7 d of treatment with 300 mM saline-alkaline mixed solution. Distilled water was used as control. Values represent averages of four independent measurements, and error bars represent standard errors. Asterisks denote a significant difference compared with the control (*$P < 0.05$)
